# Development of Mental Toughness among Basketball Sports School Students

**DOI:** 10.3390/bs14040314

**Published:** 2024-04-11

**Authors:** Eimantas Pocius, Romualdas Malinauskas

**Affiliations:** Department of Physical and Social Education, Lithuanian Sports University, LT-44221 Kaunas, Lithuania

**Keywords:** mental toughness skills, intervention program, student athletes, sport school

## Abstract

The development of mental toughness in student athletes within sports schools is crucial for identifying strengths and improving weaknesses to optimize performance. This study aimed to assess the effectiveness of a mental toughness development program for basketball sports school students. Sixty-two male student athletes, aged 15.83 ± 0.37 years, participated, with 30 in the experimental group and 32 in the control group. They completed the Mental Toughness Questionnaire 48 (MTQ48) before and after the 6-week intervention program. Pearson’s correlations were calculated for study variables. A repeated measures MANOVA followed by one-way ANOVA analyzed differences in mental toughness skills between groups and over time. Results showed a significant effect of the intervention program on mental toughness skills, with small and medium effect sizes. Post-program, the experimental group exhibited higher levels of various skills compared to controls, including skills related to challenge, commitment, emotional control, life control, overall control, self-confidence in interpersonal interactions, self-confidence in one’s abilities, overall self-confidence, and total MTQ48. These findings underscore the utility of interventions for enhancing mental toughness among basketball sports school students, emphasizing the importance of tailored approaches in such intervention programs.

## 1. Introduction

Mental toughness, defined as the personal ability to consistently pursue high levels of subjective (personal goals or aspirations) or objective outcomes (optimal performance), despite daily challenges and stressors, and the capacity to ignore significant difficulties [1], is often seen in this and other definitions of mental toughness as a term associated with positive personality resources that are highly significant in various achievement contexts. Indeed, research data highlight that athletes with higher levels of mental toughness skills demonstrate better sports outcomes [2]. Athletes with greater mental toughness cope better with stress and anxiety during competitions [3] and perform motor tasks better and with higher quality in stressful situations [4]; such athletes are more dedicated to sports [5] and are more optimistic during sports activities [6]. Athletes with mental toughness skills can much more easily overcome challenges not only in sports but also in various activities and life situations [7]. This enables us to focus not only on the specific training of athletic mental toughness skills but also on the development of universal mental toughness skills that are more adaptable to various life situations. Indeed, according to researchers [8], mental toughness is important not only for achieving high-level sports results but also for fostering socially acceptable (positive) behavior. It is stated that the training of mental toughness skills contributes to athletes’ sense of social responsibility, willingness to contribute to communal well-being, positive social integration into communal activities, flexible and positive thinking, collaborative skills, and learning from others [9]. Adolescents who are trained in mental toughness skills tend to resist the urge to engage in risky behavior [10]. The application of mental toughness training programs to athletes is particularly important as it ensures the development of athletes’ personal worth and sporting intellect [5]. Training in mental toughness skills helps athletes develop communication skills [11], contributes to athletes’ feelings of hope and self-belief, helps to better manage emotions, and fosters athletes’ resilience to trying new things in life [12]. Through mental toughness training programs, athletes’ psychological skills are developed, enhancing athletes’ well-being [13]. It is important that mental toughness training can strengthen athletes’ positive attitudes towards sports [14]. Indeed, researchers [9,15] increasingly suggest that mental toughness training should become the subject of positive youth development paradigm studies, conducting research on mental toughness training not only with adults or student athletes who are most commonly studied [15,16] but also with adolescent athletes. Therefore, this underscores the idea of cultivating mental toughness skills that are not only important in sports but also contribute to positive adolescent behavior.

Currently, the most widely preferred model of mental toughness skills across various fields is the 4C model of mental toughness, also known as the general mental toughness skills model, proposed by Clough et al. [17]. This model is based on Kobasa’s resilience model [18]. The general mental toughness model consists of skills such as commitment, challenge, control, which is further classified into life control and emotional control, and confidence, which is also further classified into self-confidence in interpersonal interactions and self-confidence in one’s abilities [17]. This model was developed because, unlike Kobasa’s resilience model, mental toughness can be measured as a specific set of skills, which is important in creating training programs that facilitate individuals’ achievements in various contexts [19].

It is crucial not to overlook scientific research data indicating that female athletes exhibit lower levels of mental toughness [16,20,21,22,23,24] and that men and women may require different numbers of sessions or even different educational methods to develop a particular skill. For instance, research data suggest that male athletes demonstrate higher levels of self-confidence in skill acquisition, and they find it easier to develop this skill [17]. Research data also indicate [25] that there may be differences in the mental toughness skill development between men and women through sports, especially in team sports, due to differences in the socialization processes of men and women when learning skills together with others. The potential for developing mental toughness skills differs between genders due to differences in hormonal regulation, which could also influence the rate of development of mental toughness skills [26]. This means that programs to build mental toughness skills should take into account existing gender differences. In addition, men’s basketball is the most popular and dominant sports branch in the country, while women’s basketball only has very few sports schools. Therefore, it was chosen to study only students at male-student basketball schools.

It is stated that deliberate training of mental toughness skills is highly effective when done through sports [27]. Indeed, three main factors are identified that facilitate the opportunities for developing mental toughness skills: (1) motivational attitudes, (2) external support, and (3) enabling experiences for improvement [28,29]. In this case, sports also provide an environment where these factors are ensured: (1) sports typically entail motivational attitudes (e.g., challenges, striving for mastery), (2) athletes are usually supported by family and fans (social support and encouragement help increase self-confidence), (3) sporting achievements are impossible without improvement (e.g., skills related to commitment to achieving goals; skills related to control, opportunities to demonstrate and enhance skills, competition). The success of skills development in sports depends on the amount of time an individual devotes to it, as well as the extent to which the specific sport is structured and whether the individual engages voluntarily [30]. Millions of adolescents worldwide and in many countries engage in sports, making it one of the most popular activities among active youths [31,32]. By participating in team sports, adolescent athletes learn to work hard, overcome failures and challenges, and collaborate with others [33]. Therefore, it can be assumed that the training of mental toughness skills will be successful if implemented in sports schools, as adolescents typically choose to attend sports schools voluntarily, and the activities conducted in sports schools are structured and involve the teaching of various sports disciplines, each with its own set of rules. Indeed, research data [34] have shown that adolescents who attended sports schools or clubs demonstrated higher levels of various skills compared to those who chose other activities. However, there are also data indicating that athletes exhibit sports-related negative behaviors [35]. Therefore, although sports are one of the best environments for skills development, it does not mean that young athletes acquire skills automatically [32], highlighting the importance of purposefully fostering athletes’ skills during sports activities.

Various skills development programs for adolescents in Lithuania are oriented towards physical education classes. Paradoxically, although adolescents actively choose to attend sports schools, participation in physical education classes in Lithuania is low. Therefore, the integration of various skills development programs into physical education classes may not ensure sufficient skills development. Meanwhile, as mentioned, adolescents choose to participate in sports schools themselves, so we can expect better results in skills development from them. Currently, there is no model-based mental toughness skills development program proposed and implemented in Lithuania that could be integrated into physical education classes in schools or sports schools. The term “model-based mental toughness skills development program” refers to a structured approach to enhancing mental toughness using a theoretical framework—the 4Cs model (control, commitment, challenge, and confidence) [17,19]. These dispositional attitudes (control, commitment, challenge, and confidence), which underpin the concept of mental toughness, may motivate young athletes to respond to stressors in a specific way, i.e., by making more effort to cope with stress and difficulty in social situations [17,19].

Based on the fact that adolescents in Lithuania are more engaged in sports by choosing sports schools, and considering that men’s basketball is the most popular and dominant sports branch in Lithuania, it is particularly important that mental toughness skills be developed through sports in basketball sports schools, using a model-based mental toughness skills development program. The aim of this study is to ascertain the impact of a model-based mental toughness skills training program on young athletes attending sports schools. We hypothesize that the development of mental toughness skills for young athletes will be effective if conducted in sports schools using a model-based mental toughness skills training program.

This study may fill a particular scientific gap because the scientific community has not yet reached a consensus on whether mental toughness can be cultivated through intervention programs, as some researchers believe [28,36] that intensive and structured sports activities naturally foster athletes’ mental toughness. In this study, we present data on the impact of a mental toughness skills development program on adolescent athletes in basketball sports schools, which are beneficial for better understanding the effects of intervention programs on the development of mental toughness skills.

## 2. Materials and Methods

### 2.1. Study Design, Study Participants, and Procedure

The sample size was determined using G* Power 3.1.9.6 software [37], taking into account a 5% α-error level and a 20% β-error level. The analysis indicated that at least 30 students for each group were required. The participants were selected by a three-stage random sampling procedure. The number of basketball sports schools required for the study was selected by lot from a list of basketball sports schools (first stage of sampling). All sports school students in the 15–16 age group of the selected basketball sports schools (second stage of sampling) were then randomly assigned to one of four groups. Finally, two of the four groups were randomly assigned (by lot) to the experimental group and two to the control group (third stage of sampling) [38,39]. A total of 62 sports school students from 2 sports schools were involved in the educational experiment: 30 in the experimental group and 32 in the control group. The mean age of the subjects at the beginning of the study was 15.83 ± 0.37 years. The similarities of the participants in each cluster were taken into account. All coaches had a similar qualification category—first-second (coaches train cadets for the national league). All teams were at a similar level, participating only in the national championship. Inherent mental toughness which may influence the results was not analyzed and it was stated as a limitation.

Participants completed questionnaires at the outset of the educational trial, prior to engaging in mental toughness development sessions, and upon its conclusion. Measures were taken to uphold the confidentiality and anonymity of research data, with questionnaires designed to collect information without identifying participants personally. Approval for the study was granted by the Social Research Ethics Committee of the Lithuanian Sports University (approval no.: SMTEK-47), and permissions were secured from the relevant sports school administrations. The questionnaire encompassed details about the study, a consent statement, demographic queries (pertaining to age), and one validated tool for gauging mental toughness indicators utilized in Lithuania.

### 2.2. Instrument

The Mental Toughness Questionnaire 48 (MTQ48) [17] was employed to evaluate general mental toughness skills. This questionnaire consists of 48 items and encompasses six scales. The challenge scale comprises nine questionnaire items (i.e., “challenges usually bring out the best in me”), while the commitment scale comprises ten (i.e., “I don’t usually give up under pressure”). Life control consists of seven questionnaire items (i.e., “whenever I try to plan something, unforeseen factors usually seem to wreck it”) and emotional control consists of seven questionnaire items (i.e., “when I am upset or annoyed, I usually let others know”). The composite measure of overall control is computed. Similarly, the composite measure of overall self-confidence is computed using two additional scales: self-confidence in interpersonal interactions, comprising six questionnaire items (i.e., “if I feel somebody is wrong, I am not afraid to argue with them”), and self-confidence in one’s abilities, comprising nine questionnaire items (i.e., “however bad things are, I usually feel they will work out positively in the end”). Additionally, a composite measure known as total mental toughness is also computed. Each questionnaire item is rated on a five-point Likert scale: 1, strongly disagree; 2, disagree; 3, neither agree nor disagree; 4, agree; 5, strongly agree [17]. This questionnaire has been adapted in Lithuania for young athletes (15–16 years old) [40]. The questionnaire’s consistency (Cronbach’s alpha coefficient = 0.79) and its subscales (Cronbach’s alpha = 0.76–0.82) were deemed satisfactory [40]. In this study, the internal questionnaire’s consistency was assessed by Cronbach’s α coefficients. To confirm the composite reliability of the measurement tool, we conducted a reliability analysis using McDonald’s ω coefficients. The consistency and composite reliability of the overall questionnaire (α = 0.85, ω = 0.88) were good. In this study, the following acceptable (adequate) internal consistency and composite reliability values were determined for the scales: challenge (α = 0.77, ω = 0.81), commitment (α = 0.82, ω = 0.83), emotional control (α = 0.71, ω = 0.72), life control (α = 0.73, ω = 0.75), self-confidence in one’s abilities (α = 0.77, ω = 0.79), self-confidence in interpersonal interactions (α = 0.74, ω = 0.75).

### 2.3. Intervention Program

To assess the efficacy of the intervention program, we employed an educational experiment method [38,39]. As the recommended average duration of mental toughness training programs typically ranges from 6 to 10 weeks [41], the educational experiment was conducted from 23 January 2023 to 10 March 2023, spanning 6 weeks. All basketball teams participating in this study played in the same national cadets championship. Since only two months had passed since the start of the season and only a few matches had been played, it can be assumed that all teams had a similar starting position in terms of season results at the time of the pre-test. The post-test was administered during the middle of the season to avoid high-pressure periods, such as play-offs or important matches, which may affect the perception of the athletes’ mental toughness.

Each specific skill was addressed in a single 60 min session. The frequency of mental toughness training sessions, based on the work of other authors, was set at once per week [42]. The intervention program commenced with an introductory activity where sports school students were introduced to the program’s goals, objectives, session rules, and other organizational matters. Additionally, activities within the intervention program were designed to cultivate various skills related to mental toughness dimensions: challenge, commitment, control (including emotional control and life control), and confidence (both self-confidence in one’s abilities and self-confidence in interpersonal interactions). Consequently, the intervention program comprised 6 sessions (one hour each). These activities were implemented with participants from the experimental group. Participants in the control group did not receive the activities outlined in the intervention program. The activities in the educational program were led by the researchers, along with the athletes’ coaches, who supported the young athletes during the activities in line with recommendations for the successful development of mental toughness skills [29]. Mental toughness skills were developed at the beginning of the training sessions. The training of mental toughness skills followed four stages: (1) skill description, (2) skill demonstration, (3) skill practice, and (4) consolidation of acquired skills [43,44].

Table 1 provides more information about the skills, objectives, and methods planned in the mental toughness training program. Various training methods were selected for developing each mental toughness skill. The training methods for each of the skills developed were research-based [14,37]. For example, to cultivate the skill related to challenge, explanation and description methods were chosen, where the skill was introduced and examples illustrating the skills were provided. The completing homework method was aimed at applying the skill in different settings—sports school students had to teach their family members how to utilize the imagery method in other life situations and describe their experience in a diary. The content of mental toughness skill training not only includes specific skill acquisition activities but also emphasizes the description and explanation of the meaning of each of these skills [45,46]. As mental toughness development programs often do not have enough time to cover a wide range of topics comprehensively, there is a need to decide and prioritize some “challenges” over others. As can be seen in Table 1, we chose to focus on injury-related challenges, but not on the challenges of learning skills or coping with loss, as injuries can be common in sports and can have a significant impact on an individual’s performance [47]. Detailed descriptions of the examples of the intervention program are attached as Supplementary Materials S1.

### 2.4. Statistical Data Analysis

The quantitative data of the study were analyzed using IBM SPSS Statistics version 28.0, a statistical package for social sciences. Descriptive statistics of the sample were computed, including arithmetic means (*M*) and standard deviations (*SD*). To assess data normality, skewness and kurtosis coefficients were evaluated. Generally, skewness and kurtosis values between −1 and 1 indicate normal data distribution [48]. The independent samples *t*-test was applied to confirm the validity of the educational experiment. Pearson’s correlations (two-tailed) were calculated for all variables. To explore variances in mental toughness skills concerning the interactions between Group (experimental and control) and Time (pre-experiment and post-experiment), a repeated measures (RM) MANOVA with a 2 (Group) × 2 (Time) design was conducted, followed by a subsequent one-way ANOVA. The effect sizes of the intervention program were assessed using partial eta-squared (*η*_p_^2^). An *η*_p_^2^ value between 0.02 and 0.12 is considered small, between 0.13 and 0.25 is medium, and greater than 0.26 is large [49].

## 3. Results

In Table 2, descriptive statistics of the overall sample’s mental toughness skills before and after the educational experiment are presented (means, *SDs*, skewness and kurtosis, and SEs of skewness and kurtosis). We can observe that the obtained data confirm the normal distribution of the data both before and after the educational experiment, as the skewness values (ranging from −0.53 to 0.73) and kurtosis values (ranging from −0.54 to 0.93) do not exceed the interval from −1 to 1.

After confirming the normal distribution of the data, we were able to apply the Student’s *t*-test and RM MANOVA. Additionally, we calculated Pearson’s bivariate correlation coefficient. The Pearson’s bivariate correlation coefficients between variables are presented separately for the time before the experiment and the time after the experiment in Table 3. These coefficients were computed to explore the associations between each dependent variable while considering multicollinearity. As none of the correlations surpassed 0.85, the multicollinearity assumption remained valid [50,51]. Mauchly’s sphericity test was satisfied because the repeated measures have only two levels.

Applying the independent samples Student’s *t*-test before the educational experiment, we found that the indicators of the skills in both experimental and control groups did not differ significantly across all measured scales, which allowed us to confirm the validity of the educational experiment: challenge (*t*_(60)_ = 0.03 ; *p* = 0.97); commitment (*t*_(60)_ = 0.11 ; *p* = 0.91); emotional control (*t*_(60)_ = −0.27 ; *p* = 0.79); life control (*t*_(60)_ = 0.04 ; *p* = 0.97); overall control (*t*_(60)_ = −0.004 ; *p* = 1.0); self-confidence in one’s abilities (*t*_(60)_ = 0.62 ; *p* = 0.54); self-confidence in interpersonal interactions (*t*_(60)_ = 0.17 ; *p* = 0.87); overall self-confidence (*t*_(60)_ = 0.51 ; *p* = 0.61); total MTQ48 (*t*_(60)_ = 0.03 ; *p* = 0.98).

To explore the impact of a mental toughness training program on the development of mental toughness skills among basketball sports school students, we conducted a repeated measures MANOVA with a 2 (Group) × 2 (Time) design. This analysis indicated a significant interaction effect of time and group membership (Wilks’ Lambda = 0.72; *F* (8.53) = 2.60; *p* < 0.05; *η^2^* = 0.28; *p* = 0.88). Analyzing the statistical indicators of the interaction between group membership and time for the mental toughness skills of the study groups (Table 4), a statistically significant effect of the training program was found on the students’ commitment (*p* < 0.05), challenge (*p* < 0.05), life (*p* < 0.05) and emotional (*p* < 0.05) control, overall control (*p* < 0.01), self-confidence in interpersonal interactions (*p* < 0.05), self-confidence in one’s abilities (*p* < 0.05), overall self-confidence (*p* < 0.01), and MTQ48 skills (*p* < 0.01) development (Table 4). 

## 4. Discussion

The data obtained from the developmental experiment allowed us to confirm our hypothesis that the training of mental toughness skills among basketball athletes in sports schools would be effective if they were trained using a model-based mental toughness skills intervention program in sports schools. After the mental toughness skills intervention program, the participants in the experimental group demonstrated statistically significant results compared to the participants in the control group on these scales: commitment (a small effect: *η*_p_^2^ = 0.100), challenge (a small effect: *η*_p_^2^ = 0.074), life (a small effect: *η*_p_^2^ = 0.067) and emotional (a small effect: *η*_p_^2^ = 0.092) control, overall control (a small effect: *η*_p_^2^ = 0.112), self-confidence in interpersonal interactions (a small effect: *η*_p_^2^ = 0.100), self-confidence in one’s abilities (a small effect: *η*_p_^2^ = 0.097), overall self-confidence (a medium effect: *η*_p_^2^ = 0.151), and total MTQ48 (a medium effect: *η*_p_^2^ = 0.200).

Our study’s findings partially align with those of other researchers, who have carried out studies with adolescent athletes. For instance, Sheard and Golby conducted a study in 2006 [52] aimed at investigating the impact of a psychological skills intervention program designed for swimmers. The psychological skills training program was tailored for adolescents aged 10–18 years old. The program consisted of 45 min sessions (once a week) attended by the participants for 7 weeks. The authors showed that the intervention program had a statistically significant impact on commitment (effect size was medium; *d* = −0.789), self-confidence (effect size was medium; *d* = −0.615), and control (effect size was small; *d* = −0.246) development. The psychological skills training also targeted the development of the skills related to challenge; however, the change in results of these skills of the interventional group was not statistically significant. We think that skills related to challenge may have been particularly sensitive in the relatively young age of the swimmers (13 years) [52], who may not be old enough to experience the character-building intervention program effects. When considering the results of the present study, the successful development of skills related to challenge as a result of the intervention program may be the use of breathing and imagery techniques. Relaxation breathing techniques can help individuals cope with the psychological challenges of injury, such as fear, frustration, and uncertainty [53]. The combination of relaxation (breathing) and imagery techniques into one procedure should effectively help the athlete to cope with the challenges [54].

Bhambri, Dhillon, and Sahni [46] examined the impact of psychological skills training on table tennis players aged 12–17 years old. The researchers provided psychological skills training to young athletes for 2 weeks, once a week. Mental toughness skills training interventions focusing on self-confidence were implemented for young table tennis players. Following the intervention, researchers indicated that participants in the experimental group exhibited statistically significantly higher indicators of self-confidence (effect size was medium) and mental toughness skills compared to young table tennis players in the control group who did not receive the intervention. The study by Bhambri et al. [55], similar to the present study, used a combined intervention consisting of relaxation (breathing) and imagery techniques and such a combination had a great effect on mental toughness skills.

Another study examining the effects of a mental toughness training program [56] was conducted with adolescent athletes (18 years old) in track and field. Participants in the experimental group attended mental toughness sessions three times a week for 8 weeks. The duration of each session was not detailed in the published article. After the completion of the educational experiment, the researchers showed that the mental toughness training program had a statistically significant impact on the self-confidence (effect size was large; *d* = −1.989) of the athletes in the experimental group compared to the control group of track and field athletes. As the intervention methods were similar to those in the current study, it can be assumed that the higher effect size than in the present study is due to the fact that the program lasted two weeks longer and was delivered more times per week (three times). Successfully fostering the mental toughness of young soccer players (average age 14.58 years) through a mental toughness training program was achieved by Gucciardi, Gordon, and Dimmock [57]. After the educational experiment, all trained athletes in the experimental group exhibited statistically significantly higher mental toughness skills compared to the control group: commitment (a large effect: *η*_p_^2^ = 0.55), control (a large effect: *η*_p_^2^ = 0.38), and challenge (a medium effect: *η*_p_^2^ = 0.18). The duration of the applied mental toughness training program was 6 weeks, with sessions lasting 2 h conducted once a week. We consider that the longer sessions (2 h) may explain the fact that the effect sizes for some of the indicators were stronger than in the present study.

The impact of a mental toughness training program was also evaluated in the context of non-athletic adolescents by Kiarostami, Jeddi, Roohinezhad, and Hakimi [58]. A program lasting seven weeks, consisting of two-hour sessions per week, was found to be statistically significantly effective for the experimental group participants in developing skills related to challenge (a medium effect: *η*_p_^2^ = 0.246), commitment (a large effect: *η*_p_^2^ = 0.380), emotional control (a large effect: *η*_p_^2^ = 0.297), life control (a large effect: *η*_p_^2^ = 0.278), self-confidence in one’s abilities (a large effect: *η*_p_^2^ = 0.256), and self-confidence in interpersonal interactions (a large effect: *η*_p_^2^ = 0.273) compared to the control group of adolescents. The larger effect size may again be due to the same explanation, that the sessions lasted longer (two-hour sessions) than in the present study. In summary, various research data confirm that mental toughness can be successfully developed when mental toughness skills intervention programs are employed. Different scientific studies have observed varying results in skill development (effect sizes), which may vary due to the different numbers of sessions and durations of the applied mental toughness skill intervention programs.

The potential mechanisms of the intervention’s training methods on mental toughness-related constructs help us to better understand the benefits of the intervention program. For instance, the combination of relaxation (breathing) and imagery techniques into one procedure should be beneficial for the athlete as he/she has to psychologically train for a specific situation under simulated situation conditions [54]. Mentally rehearsing or reliving the experience of the situation (for instance, injury or competition) should help the athlete to cope with the challenges. The feedback method encourages regular feedback mechanisms and self-reflection practice [54]. By receiving positive feedback, a growth mindset is fostered, allowing young athletes to continuously improve their skills and adapt to changing circumstances, thus promoting resilience and adaptability in both sport and life [27]. Mental toughness training usually involves cognitive restructuring techniques aimed at altering negative thought patterns and promoting more adaptive beliefs and attitudes. This can enhance feelings of control by empowering athletes to manage their emotions and perceptions more effectively [27].

Therefore, it is reasonable to suggest that mental toughness skills measured by the MTQ48 are related to other factors relevant to sport and other social interaction situations (for instance, motivational processes) because the combination of these skills related to “dispositional attitudes (challenge, commitment, control, confidence) is thought to motivate one to respond to stressors with specific coping and social interaction efforts which facilitate resiliency by turning potential disasters into opportunities” ([59], p. 332). The advancement of mental toughness skills holds significant implications for sports specialists and coaches in practical settings, as dispositional attitudes play a pivotal role in the shaping of thoughts, feelings, actions, and convictions, thereby aiding the adjustment to the surrounding environment [60].

### Study Strengths and Limitations

The effective mental toughness skills intervention program presented in this study is the strongest aspect of our research. Future researchers will be able to integrate the skills comprising the model into studies of positive youth development, aiming at fostering positive behavior among adolescents.

One of the limitations of this study is that it only included young male sports school students. One reason why only male athletes were chosen for the study is gender-related psychological and existing social differences that influence the expression of mental toughness and learning characteristics between males and females. Another reason why only male athletes were chosen for the study is related to the popularity of men’s basketball in Lithuania. This results in fewer young female basketball players attending sports schools, and only a few sports schools in the country have groups of young female basketball players. Nonetheless, future researchers could conduct studies that explore the mental toughness development characteristics of young female athletes.

Another limitation of this study is that it involved representatives of one sports discipline. This limitation is related to sociocultural aspects, where basketball is the most popular sport in Lithuania. Directing research towards a larger variety of sports disciplines would result in non-homogeneous research samples, and it would also be challenging to conduct studies with participants from other sports disciplines. This could be due to the limited number of sports schools for certain sports, which would restrict the possibility of conducting a probabilistic selection. Additionally, the activities of skill development programs should be tailored to the specifics of other team sports because some of the activities are specific to basketball (see Supplementary Materials S1). In future research, intervention programs and their content should be adapted to the specifics of other team or individual sports disciplines.

A potential limitation of the study could be the failure to take into account the inherent mental toughness of the players, which may have influenced the results. This should therefore be considered in further studies. The reliance on self-reported instruments serves as one more potential limitation in this study. Consequently, in future, researchers evaluating the effectiveness of psychological interventions must collect both subjective and objective data when such relationships exist [52].

## 5. Conclusions

The data from this study helped address certain existing scientific gaps related to the peculiarities of mental toughness skills development among young athletes. Firstly, in this study, we developed and proposed to the scientific community a skills-based mental toughness training program tailored for young athletes. Secondly, it is also important to note that most of the mental toughness development programs proposed by researchers are oriented toward adults and high-performance athletes, while our proposed program is intended for adolescent sports school students. During this research, we found that a model-based, 6-week mental toughness skills intervention program had a significant impact on basketball sports schools’ students’ skills related to commitment, challenge, life control, emotional control, overall control, self-confidence in interpersonal interactions, self-confidence in one’s abilities, overall self-confidence, and total MTQ48 mental toughness skills.

These conclusions can be beneficial for future researchers who will examine issues related to the training of mental toughness skills or integrate the training of mental toughness skills into studies on positive youth development. Additionally, these findings may encourage future researchers to conduct more studies focused specifically on the adolescent period, which has been relatively underexplored. The data obtained during the study allow for a better understanding of the benefits of training mental toughness skills, challenging the notion that intensive and structured sports activities inherently develop mental toughness skills.

## Figures and Tables

**Table 1:** Description of a mental toughness skills training program for young basketball players in sports schools.

Intervention	Number of Sessions	Content	Goals	Training Methods
General mental toughness skills	1	Challenge	Learn to see injuries as a challenge that simply needs to be accepted and overcome.	Explanation, description, breathing method, imagery method, completing homework.
	1	Commitment	Learn to achieve sporting goals and to manage one’s reactions to victory or defeat.	Explanation, description, discussion giving feedback, case study, completing homework.
	2	Control	Learn to sufficiently control the factors that influence behavior to achieve their goals and to manage their emotions and disclose their emotional state to others.	Explanation, description, giving feedback, breathing method, imagery method, case study, completing homework.
	2	Confidence	Learn to understand that he/she can meet the challenges that life presents and that he/she presents to himself/herself, and to interact (communicate, cooperate, compete) with others when interacting with others in a way that feels good (comfortable) and is able to be decisive and assertive when needed.	Explanation, description, discussion, case study, method of active games, giving feedback, completing homework.

**Table 2:** Means (*M*), standard deviations (*SD*), and normality tests of the study variables (*N* = 62).

		Before Experiment					After Experiment					
	*M*	*SD*	Sk	SkSE	Ku	KuSE	*M*	*SD*	Sk	SkSE	Ku	KuSE
Challenge	3.62	0.41	0.46	0.30	0.16	0.60	3.75	0.35	0.30	0.30	0.24	0.60
Commitment	3.39	0.29	−0.05	30	−0.39	0.60	3.51	0.36	0.21	0.30	−0.23	0.60
Emotional control	3.08	0.32	0.55	0.30	0.59	0.60	3.19	0.38	−0.29	0.30	0.55	0.60
Life control	3.27	0.37	0.18	0.30	−0.40	0.60	3.38	0.37	−0.42	0.30	0.93	0.60
Overall control	3.17	0.29	0.49	0.30	0.58	0.60	3.29	0.04	0.73	0.30	0.37	0.60
Self-confidence in one’s abilities	3.36	0.39	−0.19	0.30	−0.54	0.60	3.45	0.37	0.51	0.30	−0.12	0.60
Self-confidence in interpersonal interactions	3.20	0.30	−0.53	0.30	0.42	0.60	3.31	0.32	−0.18	0.30	0.07	0.60
Overall self-confidence	3.28	0.29	−0.44	0.30	0.24	0.60	3.38	0.27	0.37	0.30	0.36	0.60
Total MTQ-48	3.37	0.26	0.55	0.30	0.83	0.60	3.49	0.23	0.38	0.30	−0.43	0.60

Sk—Skewness; SkSE—Skewness standard error; Ku—Kurtosis; KuSE—Kurtosis standard error.

**Table 3:** Correlations of dependent variables.

	Challenge	Commitment	Emotional Control	Life Control	Overall Control	Self-Confidence in One’s Abilities	Self-Confidence in Interpersonal Inter-Actions	Overall Self-Confidence	Total MTQ-48
Challenge	1	0.402 **	0.101	0.489 **	0.433 **	0.429 **	0.373 **	0.510 **	0.796 **
2. Commitment	0.449 **	1	0.187 *	0.069	0.192 *	0.300 **	0.340 **	0.403 **	0.699 **
3. Emotional control	0.213 *	0.169	1	−0.094	0.688 **	0.274 **	0.186 *	0.296 **	0.393 **
4. Life control	0.570 **	0.180 *	0.091	1	0.658 **	0.195 *	0.099	0.191 *	0.449 **
5. Overall control	0.523 **	0.234 **	0.736 **	0.725 **	1	0.349 **	0.213 *	0.362 **	0.624 **
6. Self-confidence in one’s abilities	0.471 **	0.325 **	0.358 **	0.328 **	0.471 **	1	0.243 **	0.824 **	0.639 **
7. Self-confidence in interpersonal interactions	0.435 **	0.331 **	0.167	0.196 *	0.236 **	0.331 **	1	0.751 **	0.588 **
8. Overall self-confidence	0.556 **	0.401 **	0.333 **	0.328 **	0.447 **	0.844 **	0.772 **	1	0.779 **
9. Total MTQ-48	0.841 **	0.685 **	0.439 **	0.587 **	0.694 **	0.689 **	0.592 **	0.789 **	1

Correlations below the diagonal are for Time before experiment. Correlations above the diagonal are for Time after experiment. *N* = 62. * *p* < 0. 05; ** *p* < 0.01.

**Table 4:** Mean scores of mental toughness skills of young basketball players in sports schools before and after the educational experiment.

	Experimental Group *M* (*SD*)		Control Group *M* (*SD*)		Univariate Tests of RM MANOVA Group × Time		
Social-Emotional Skills Related to:	Before Experiment	After Experiment	Before Experiment	After Experiment	*F*_1.60_	*p*	*η*_p_^2^
Challenge	3.63 (0.39)	3.89 (0.32)	3.61 (0.43)	3.62 (0.32)	4.78 *	0.033	0.074
Commitment	3.39 (0.29)	3.63 (0.40)	3.39 (0.29)	3.39 (0.28)	6.68 *	0.012	0.100
Emotional control	3.07 (0.31)	3.31 (0.35)	3.09 (0.33)	3.08 (0.38)	6.01 *	0.017	0.092
Life control	3.28 (0.38)	3.51 (0.32)	3.27 (0.37)	3.26 (0.38)	4.32 *	0.042	0.067
Overall control	3.17 (0.30)	3.41 (0.22)	3.17 (0.29)	3.17 (0.23)	7.59 **	0.008	0.112
Self-confidence in one’s abilities	3.39 (0.41)	3.62 (0.37)	3.33 (0.38)	3.29 (0.30)	6.45 *	0.014	0.097
Self-confidence in interpersonal interactions	3.21 (0.31)	3.44 (0.30)	3.19 (0.30)	3.20 (0.29)	6.69 *	0.012	0.100
Overall self-confidence	3.30 (0.30)	3.53 (0.25)	3.26 (0.29)	3.25 (0.22)	10.69 **	0.002	0.151
Total MTQ-48	3.37 (0.24)	3.62 (0.21)	3.37 (0.28)	3.36 (0.17)	14.96 **	0.000	0.200

*η*_p_^2^—partial eta squared; * *p* < 0.05; ** *p* < 0.01.

## Data Availability

The datasets collected and analyzed during the current study are available from the corresponding author on reasonable request.

## Supplementary Materials

The following supporting information can be downloaded at: https://www.mdpi.com/article/10.3390/bs14040314/s1, Supplementary S1. Examples from a mental toughness skills development program; Figure S1. Specific combination. Refs. [61,62] are cited in the Supplementary Materials.
