# Supplementary material for: Development of Mental Toughness among Basketball Sports School Students
Source: Behav Sci (Basel). 2024 Apr 11;14(4):314. doi: 10.3390/bs14040314 (PMC11047309; doi:10.3390/bs14040314)
Supplement: Supplementary file 1 [file behavsci-14-00314-s001.zip › behavsci-2929729-supplementary.pdf]

behavioral sciences

MDPI

Supplementary S1

Examples from a mental toughness skills development program

1. Example from a mental toughness skills development program. Control skills development activity (1 session).

- ➢ **The aim of these activities** is to develop the control skills of sports school students.
- ✓ **Brief description.** During the activities, sports school students will become acquainted with the concept and classification of control skills, and they will perform exercises to develop these skills. To consolidate the skill, homework assignments will be given to the sports school students.
- ➢ **Training methods:** explanation, description, giving feedback, case study, completing homework.
- ➢ **Training tools:** basketball court, basketball, pens, sheets of paper.
- ➢ **--**
- **Introduction.** The coach of sports school students asks, "*What do you think control is?*" Listens to the sports school students and introduces control skills - *now let's try to reveal what control skills are and how to develop them: control skills are described as the ability to manage personal reactions in various sports contexts, the ability to concentrate on activity barriers and deal with them* [1] (<u>description of the skill</u>). *Control skills are further classified into life control and emotion control. Life control is related to a person's belief that they sufficiently control factors influencing their behavior and activities, and therefore achieve their goals* [2] (<u>description of the skill</u>*). This means that individuals with this skill believe that their plans will not be thwarted and that they can change something in their own or even others' lives. We will perform exercises aimed at developing life control skills.*
- **Presentation of exercises.** *During this task, you will critically assess your current life (in our case, sports) situation and whether everything is going as you would like (description of the skill). Now, you will divide into two teams and play one set. While playing, pay attention to what you struggle with the most.*
- **Task exercise.** When the sports school students finish playing one set and take a brief rest, the coach distributes papers and pens to them. Then, another part of the task is introduced. *Now, we will analyze ourselves, trying to get to know ourselves better. By understanding ourselves better, we can better control the processes happening in our lives. On the paper distributed, write down the letters A, B, and C. Now, next to letter A, write down what went wrong for you during the game. Remember, and next to letter B, write down how you behaved when things weren't going well? Did you get overwhelmed by negative emotions? Did you push your opponent? Or did you repeat what wasn't working for you? Finally, next to letter C, write down the consequences of your behavior (emotions, choices, actions). Did your mood not get affected? Did you not offend your opponent? Did it not happen that failures just increased and the team performed even worse?* (<u>demonstration of the skill</u>). *By identifying A-B-C factors in every challenging situation, you become stronger and deal with any stressful situation easier because you already know your behavioral patterns and possible consequences. This way, you control what happens in your life. Now, let's play the second set. Remember your identified A-B-C.* The sports school students play the second set under the coach's supervision (<u>practice of the skill</u>).

  After the sports school students finish the game, the coach initiates a discussion to provide feedback on the experiences of the sports school students: *how did your game change? Perhaps the team's mood and atmosphere improved? Did the number of mistakes decrease? Did the ability to control your actions, even when things aren't going well, contribute to an easier path towards the goal?* After the discussion with the sports school students, the coach explains that *when we understand specifically how we tend to behave when something doesn't go well or we don't like it, by changing our behavior, we feel like we're*

in control of our lives and can achieve our goals. In such a case, even a lost set doesn't evoke so many unpleasant emotions because we know that we controlled our actions and did everything we could at that moment to achieve the goal.

- **Homework presentation.** Then homework assignments are presented for <u>the consolidation of acquired skills</u>. *Controlling events and goals in our lives can be challenging simply because we often aren't deeply involved in the situation. Complete the homework assignment:*

  Make a list of things you enjoy, find useful, and give you strength:

  a) Describe how valuable and beneficial it is for you to do what you enjoy.

  b) Describe how often you do what you enjoy. Would you like to do it more often?

  Consider and describe what prevents you from doing it more often. Once you identify the reasons, eliminate them. Share your experience with your teammates.

2. Example from a mental toughness skills development program. Confidence skills development activity (1 session).

- **The aim of these activities** *is to develop the self-confidence skills of sports school students.*
- **Brief description.** During the activities, sports school students will become acquainted with the concept and additional classification of self-confidence skills, and they will perform exercises. To consolidate the skill, homework assignments will be given to the sports school students.
- **Training methods:** explanation, description, discussion, method of active games, case study, giving feedback, completing homework.
- **Training tools:** basketball court, basketball.
- ---

- **Introduction.** The coach invites sports school students to a discussion: *What do you think a sports school student needs in order to have self-confidence?* The coach engages in a discussion with the sports school students. After the discussion, the coach introduces to the sports school students what self-confidence skills are: *self-confidence skills are related to the ability to evaluate one's position within the team, the ability to assess one's capabilities, given changing environmental conditions* [1] (<u>description of the skill</u>). *These skills are further categorized into self-confidence in one's abilities skills and self-confidence in interpersonal interaction skills. During this training session, we will learn to cultivate the self-confidence in one's abilities skill. The self-confidence in abilities skill is related to an individual's understanding that they are capable of performing the tasks that life presents to them and those they set for themselves* [2] (<u>description of the skill</u>).
- **Presentation of exercises.** The coach asks sports school students: *does it happen while playing basketball that when the environmental conditions change (such as game speed, style, team composition, etc.), we start to doubt ourselves and our ability to continue playing, for example, in a different position? Definitely, yes, it does happen that when circumstances change suddenly, we become afraid and start to believe that various goals may no longer be achievable. During this task, you will reveal your other skills that are useful when the game conditions change unexpectedly (e.g., you have to replace an injured teammate in a different position). This will allow you to assess your abilities, your position within the team, feel valuable as a team member, and believe that you are capable of achieving the goals required due to changing circumstances (description of the skill). How will the task look? We will divide into 2 groups of 5 players each. First, we will practice a specific combination, with certain players taking positions they usually play. Both groups will practice this three times in a row.* The combination looks like this: Player 1 passes the ball to Player 4. At the same time, Player 3 runs to Zone A, Player 5 to Zone B, and Player 2 to Zone C. Player 4 passes the ball to Player 5 in Zone B. Player 5, considering the situation, either shoots the ball or passes it to Player 2 in Zone C (figure 1) (<u>demonstration of the skill</u>).

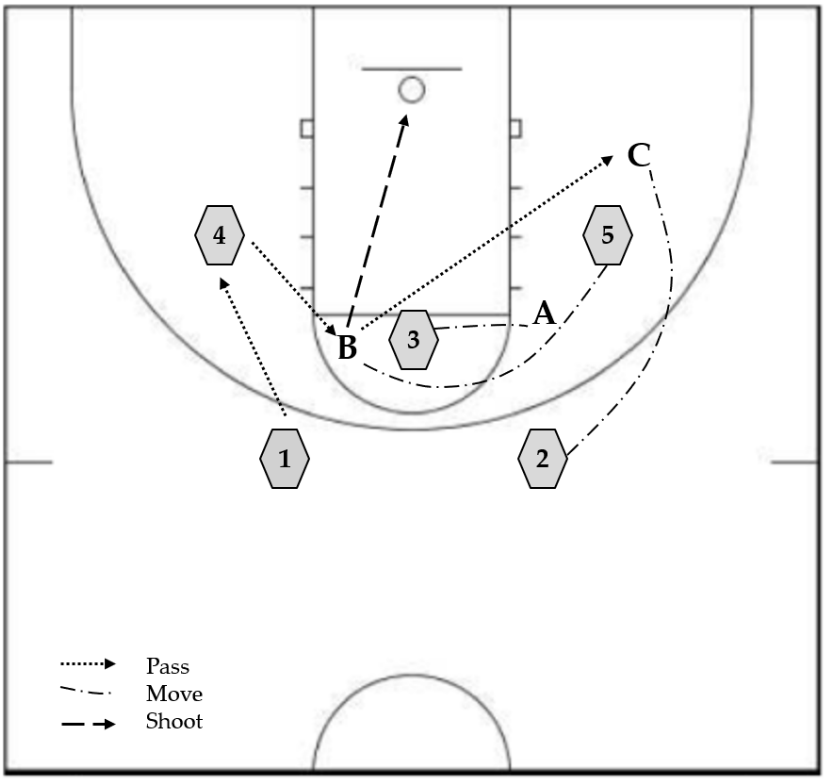

Figure S1. Specific combination.

- **Task exercise.** The sports school students perform the task. As the sports school students perform the task, the coach informs them that the group's players will now be mixed, and each position will now be occupied by a player who usually does not play in that position. Then, the groups of sports school students practice their combinations three times in a row (practice of the skill). After completing the task, the coach asks the sports school students to reflect on and analyze how they did. Then, the coach asks them: *how did it go? Did you manage to achieve the goals despite the change in the team composition (circumstances)?* After listening to the sports school students, the coach summarizes that *they were able to achieve the goals even when it seemed like they wouldn't, as their developing skills are usually intended for different tasks on the court. Nevertheless, each player has various skills that come in handy when circumstances change. Each of you is capable of doing much more on the court than you are used to doing.*
- **Homework presentation.** In all activities, it is important for us to have self-confidence. Complete your homework (consolidation of acquired skills). Analyze yourself and figure out what skills/abilities you have that can be used in other life activities. Describe how you did and share it with the coach and your teammates.

Skills development activities adapted by the authors, based on [1-4].

References

1. Clough, P.; Earle, K.; Sewell, D. Mental toughness: The concept and its measurement. *Solutions in sport psychology* **2002**, *1*, 32-43.
2. Strycharczyk, D.; Clough, P.; Perry J. *Developing mental toughness: Strategies to improve performance, resilience and wellbeing in individuals and organizations;* Kogan Page Publishers: London, New York, 2021.
3. Selk, J. *10-Minute and Executive Toughness;* McGraw Hill Professional: New York, Chicago, San Francisco, 2013.
4. Sheard, M. *Mental toughness: The mindset behind sporting achievement*; Routledge: London, New York, 2012.
